# Efficacy of mirabegron in patients with and without prior antimuscarinic therapy for overactive bladder: a post hoc analysis of a randomized European-Australian Phase 3 trial

**DOI:** 10.1186/1471-2490-13-45

**Published:** 2013-09-18

**Authors:** Vik Khullar, Javier Cambronero, Javier C Angulo, Marianne Wooning, Mary Beth Blauwet, Caroline Dorrepaal, Nancy E Martin

**Affiliations:** 1 Urogynaecology Department, St Mary’s Hospital, Imperial College, London, UK; 2 Infanta Leonor Hospital, Department of Urology, Madrid, Spain; 3 Hospital Universitario De Getafe, Department of Urology, Madrid, Spain; 4 Astellas Pharma Global Development, Global Clinical Science, Leiden, The Netherlands; 5 Astellas Pharma Global Development, Inc., Biostatistics, Northbrook, IL, USA; 6 Astellas Pharma Global Development – EU, Global Medical Science – Urology, Leiden, The Netherlands; 7 Astellas Pharma Global Development, Global Medical Sciences, Northbrook, IL, USA

**Keywords:** β_3_-adrenoceptor agonist, Mirabegron, OAB, Overactive bladder, Post hoc analysis

## Abstract

**Background:**

Antimuscarinic agents are currently the predominant treatment option for the clinical management of the symptoms of overactive bladder (OAB). However, low rates of persistence with these agents highlight the need for novel, effective and better-tolerated oral pharmacological agents. Mirabegron is a β_3_-adrenoceptor agonist developed for the treatment of OAB, with a mechanism of action distinct from that of antimuscarinics. In a randomized, double-blind, placebo- and active-controlled Phase 3 trial conducted in Europe and Australia (NCT00689104), mirabegron 50 mg and 100 mg resulted in statistically significant reductions from baseline to final visit, compared with placebo, in the co-primary end points – mean number of incontinence episodes/24 h and mean number of micturitions/24 h. We conducted a post hoc, subgroup analysis of this study in order to evaluate the efficacy of mirabegron in treatment-naïve patients and patients who had discontinued prior antimuscarinic therapy because of insufficient efficacy or poor tolerability.

**Methods:**

Patients were randomized to placebo, mirabegron 50 or 100 mg, or tolterodine extended release (ER) 4 mg orally, once-daily, for 12 weeks. For the post hoc analysis, the primary patient population was divided into the following subgroups: (1) patients who had not received any prior antimuscarinic OAB medication (treatment-naïve) and (2) patients who had received prior antimuscarinic OAB medication. The latter subgroup was further subdivided into patients who discontinued due to: (3) insufficient efficacy or (4) poor tolerability. Analysis of the co-primary efficacy endpoints by subgroup was performed using analysis of covariance with treatment group, subgroup, sex, geographical region, and subgroup-by-treatment interaction as fixed factors; and baseline value as a covariate.

**Results:**

Mirabegron, 50 mg and 100 mg once-daily, demonstrated similar improvements in the frequency of incontinence episodes and micturitions in OAB patients who were antimuscarinic-naïve and who had discontinued prior antimuscarinic therapy. While mirabegron demonstrated improvements in incontinence and micturition frequency in patients who had discontinued prior antimuscarinic therapy due to insufficient efficacy, the response to tolterodine was similar to that of placebo.

**Conclusion:**

In this post hoc subgroup analysis, mirabegron provided treatment benefits in OAB patients who were antimuscarinic treatment-naïve and in patients who had received prior antimuscarinic treatment.

## Background

Overactive bladder syndrome (OAB) affects more than 400 million people worldwide [1]. Antimuscarinic agents, such as tolterodine, are the current mainstay of pharmacotherapy for the clinical management of OAB [2-4]. However, OAB patients may have a suboptimal response to antimuscarinics or find the associated adverse events (AEs), such as dry mouth, constipation, and blurred vision [5-7], to be intolerable. Bothersome side effects and/or inadequate efficacy contribute to the low persistence rates seen with antimuscarinics [8-12]. One systematic review found rates of discontinuation of 43% to 83% within the first 30 days and discontinuation rates continuing to rise over time [10].

The limitations of antimuscarinic therapy indicate that there is a need for oral pharmacological treatment options that are both effective and well tolerated. Mirabegron, a β_3_-adrenoreceptor agonist with a mechanism of action distinct from that of antimuscarinic agents, is the first drug in this class of agents to have been approved for the treatment of the symptoms of OAB [13]. Mirabegron elicits β_3_-adrenoreceptor-mediated relaxation of the detrusor muscle during the storage phase, thereby improving bladder capacity without impeding bladder voiding [14,15]. The efficacy of once-daily mirabegron administered orally at doses of 25 mg, 50 mg, and 100 mg, in the treatment of urinary frequency, urgency, and incontinence in patients with OAB has been demonstrated in three 12-week, Phase 3 studies of mirabegron (NCT00662909, NCT00689104, and NCT00912964) [16-18]. In the European-Australian study (NCT00689104 [17]), mirabegron 50 mg and 100 mg resulted in statistically significant reductions from baseline to final visit, compared with placebo, in the co-primary end points – mean number of incontinence episodes/24 h (adjusted mean changes from baseline [95% confidence intervals (CI)] of –1.57 [–1.79, –1.35] and –1.46 [–1.68, –1.23] for mirabegron 50 mg and 100 mg, respectively, vs –1.17 [–1.39, –0.95] for placebo; p < 0.05 for comparisons of both mirabegron doses with placebo) and mean number of micturitions/24 h (–1.93 [–2.15, –1.72] and –1.77 [–1.99, –1.56] for mirabegron 50 mg and 100 mg, respectively, vs –1.34 [–1.55, –1.12] for placebo; p < 0.05 for comparisons of both mirabegron doses with placebo).

Here we present a post hoc, subgroup analysis of the European-Australian study [17] designed to assess the efficacy of mirabegron in the subgroups of patients who had not previously received antimuscarinics (treatment-naïve) and in those who had discontinued prior antimuscarinic therapy because of insufficient efficacy or poor tolerability.

## Methods

### Primary study design

**Figure 1:**
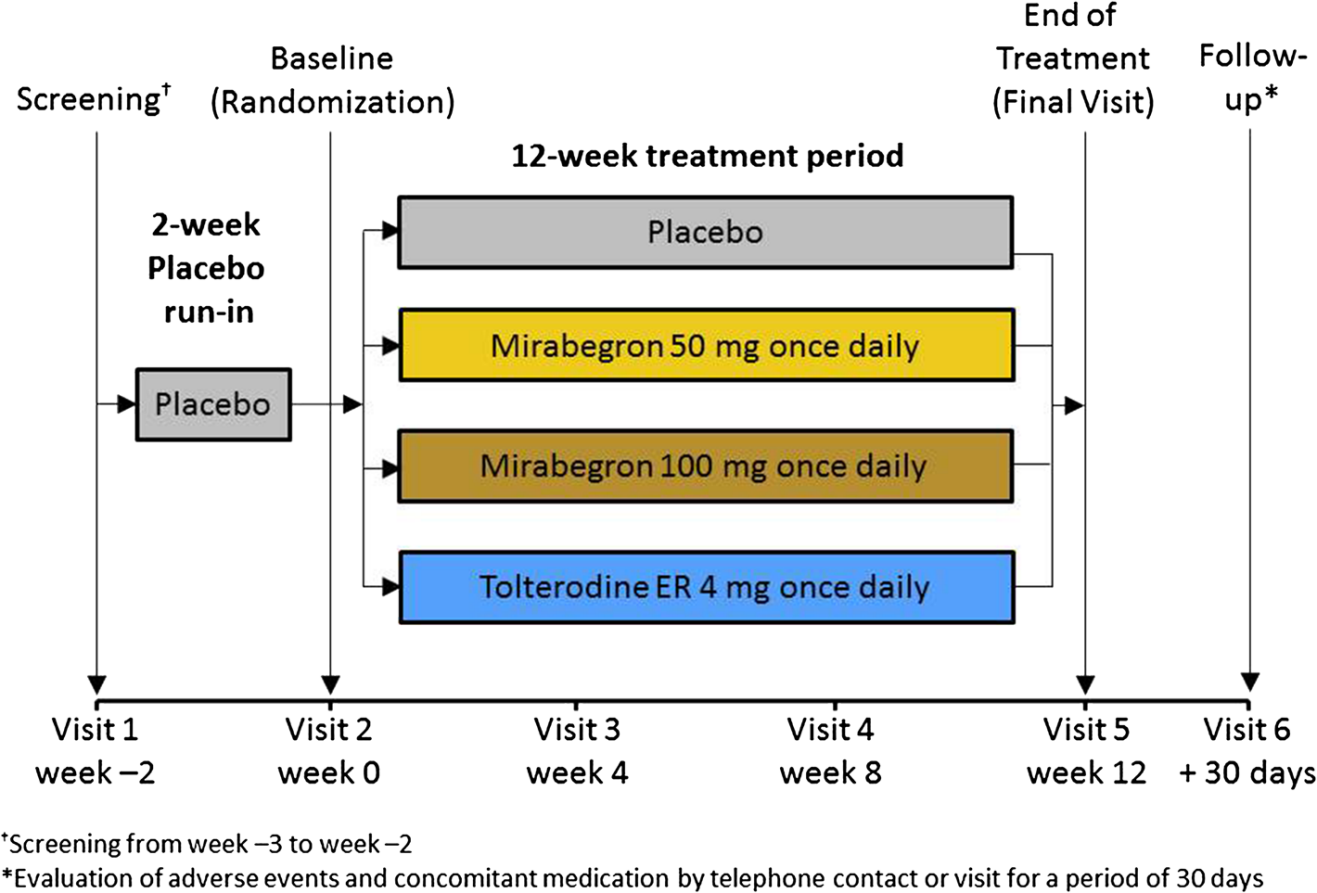
Study design.

The primary study [17] was a 12-week, multicenter, randomized, double-blind, parallel-group, placebo- and active-controlled Phase 3 trial, conducted at 189 sites in 27 countries throughout Europe and Australia. The study design has been described in detail previously [17] (Figure 1). In brief, the population consisted of men and women aged ≥18 years with symptoms of OAB for ≥3 months at screening and who had experienced an average of ≥8 micturitions/24 h and ≥3 urgency episodes (with or without incontinence) during the 3-day micturition diary period at baseline. Eligible patients were randomized (1:1:1:1) to placebo, mirabegron 50 mg, mirabegron 100 mg, or tolterodine extended release (ER) 4 mg once-daily for 12 weeks. Patients were excluded if they had an average total daily urine volume >3000 ml or suffered from stress incontinence or mixed incontinence which was stress predominant at screening.

Patients were also excluded from the study if they were taking any medications intended to treat OAB. There were no restrictions as to the number of antimuscarinic drugs that a patient could have received prior to this study. In addition, patients were not allowed to take any nondrug treatment for OAB during the study except for ongoing bladder training or pelvic floor exercise programs that had started at least 30 days prior to the start of the study. All previous and concomitant medications, including drug and non-drug treatments, taken within 30 days of screening, and any OAB medications taken at any time prior to screening, were to be recorded. The reason for the discontinuation of any prior OAB medication was to be recorded as insufficient efficacy, poor tolerability, or other. More than one reason could be checked.

The study was approved by the institutional review board at each site and conducted in accordance with the ethical principles that have their origin in the Declaration of Helsinki, Good Clinical Practice, International Conference on Harmonisation guidelines, and all applicable laws and regulations.

### Efficacy assessments and statistical analyses

The co-primary efficacy end points were change from baseline to final visit (end of treatment) in mean number of incontinence episodes and mean number of micturitions/24 h. Descriptive statistics were used for baseline demographics/clinical characteristics. Analysis sets were as follows: the safety analysis set (SAF) comprising all patients who took at least one dose of double-blind study drug; the full analysis set (FAS) comprising SAF patients who had at least one micturition measurement in the 3-day baseline diary and at least one post-baseline diary; and the FAS-incontinence (FAS-I) set comprising FAS patients who reported at least one incontinence episode in the 3-day baseline diary.

In this post hoc analysis, the primary patient population was divided into the following subgroups: (1) patients who had not received any prior antimuscarinic OAB medication (treatment-naïve) and (2) patients who had received prior antimuscarinic OAB medication. The latter subgroup was further subdivided into patients who discontinued due to: (3) insufficient efficacy or (4) poor tolerability. As patients could check any or all of: “insufficient efficacy”, “poor tolerability”, or “other” on the electronic case report form (eCRF) as reasons for discontinuation of prior antimuscarinic OAB medication, patients could be included in both the “insufficient efficacy” and “poor tolerability” subgroups. (The subgroup comprising patients who discontinued only for “other” reasons” and who therefore did not appear in either the “insufficient efficacy” or “poor tolerability” subgroups was not evaluated). Subgroup analyses were performed using analysis of covariance with treatment group, subgroup, sex, geographical region, and subgroup-by-treatment interaction as fixed factors; and baseline value as a covariate. The original study [17] was not powered to detect a statistically significant difference between treatment groups for each subgroup.

## Results

### Demographics and baseline characteristics

A total of 1987 eligible patients were randomized and 1978 patients received double-blind treatment in the primary study (the SAF population) [17]. The baseline demographic and clinical characteristics of the SAF were similar across treatment groups (Table 1). The majority were Caucasian (99.1%) and female (72.2%) with a mean (± standard deviation [SD]) age of 59.1 (± 12.6) years. The baseline demographic and clinical characteristics of the FAS (n = 1906) were similar to that of the SAF. In the FAS-I (n = 1165) the proportion of female patients (83.4%) and patients aged 65 years or older (39.6%) were higher than in the SAF or FAS populations. OAB history characteristics were comparable across the treatment groups [17]. Overall, 48.6% of patients in the FAS (Table 1) and 53.3% of those in the FAS-I had previously received antimuscarinic OAB medication, but discontinued it before study entry. Previous OAB medication was one or more of: solifenacin, oxybutynin, tolterodine, trospium, propiverine, darifenacin, or fesoterodine. Of these, solifenacin, taken by ~24% of all FAS patients and ~48% of FAS patients who had received previous OAB medication, was the most common. Tolterodine was taken by 27.2% of patients who received prior OAB medication. Insufficient efficacy was given as a reason for the discontinuation of prior treatment by 66.9% of patients in both the FAS and FAS-I populations who received prior OAB medication (Table 1) [17]. Poor tolerability was given as a reason for the discontinuation of prior treatment by 26.7% and 28.1% of patients in the FAS and FAS-I populations, respectively, who received prior OAB medication. As patients could check more than one reason for the discontinuation of prior treatment, overlap between both subgroups was possible. However, no more than 13.1% of FAS patients in any treatment group who received prior OAB medication cited both poor tolerability and insufficient effect as reasons for discontinuation.

**Table 1:** Demographic and baseline characteristics (SAF) and OAB history (FAS), by treatment group

	**Placebo**	**Mirabegron**	**Mirabegron**	**Tolterodine**
		**50 mg**	**100 mg**	**ER 4 mg**
SAF				
Patients, n	494	493	496	495
Sex, n (%)				
Female	356 (72.1)	357 (72.4)	355 (71.6)	361 (72.9)
Age group, n (%)				
≥65 years	181 (36.6)	178 (36.1)	183 (36.9)	192 (38.8)
≥75 years	44 (8.9)	46 (9.3)	46 (9.3)	37 (7.5)
Age, mean (SD)	59.2 (12.3)	59.1 (12.4)	59.0 (12.7)	59.1 (12.9)
Race, n (%)				
Caucasian	490 (99.2)	488 (99.0)	492 (99.2)	490 (99.0)
Body mass index, kg/m^2^, mean (SD)	27.8 (5.0)	27.5 (4.9)	28.0 (5.0)	27.8 (5.0)
FAS				
Patients, n	480	473	478	475
Type of OAB, n (%)*				
Urgency incontinence	201 (41.9)	192 (40.6)	179 (37.4)	184 (38.7)
Frequency	177 (36.9)	173 (36.6)	183 (38.3)	186 (39.2)
Mixed	102 (21.3)	108 (22.8)	116 (24.3)	105 (22.1)
Previous OAB medication				
Yes (any, n [% of FAS])	238 (49.6)	240 (50.7)	237 (49.6)	231 (48.6)
Solifenacin, n (%)^†^	127 (53.4)	107 (44.6)	112 (47.3)	109 (47.2)
Oxybutynin	77 (32.4)	82 (34.2)	82 (34.6)	79 (34.2)
Tolterodine	69 (29.0)	59 (24.6)	71 (30.0)	58 (25.1)
Trospium	44 (18.5)	45 (18.8)	41 (17.3)	49 (21.2)
Propiverine	22 (9.2)	23 (9.6)	16 (6.8)	17 (7.4)
Darifenacin	14 (5.9)	8 (3.3)	21 (8.9)	12 (5.2)
Fesoterodine	4 (1.7)	1 (0.4)	2 (0.8)	2 (0.9)
Reason for previous OAB medication discontinuation, n (%)^†,‡^				
Insufficient effect	159 (66.8)	160 (66.7)	159 (67.1)	155 (67.1)
Poor tolerability	68 (28.6)	65 (27.1)	64 (27.0)	56 (24.2)
Insufficient effect and poor tolerability	26 (10.9)	28 (11.7)	31 (13.1)	25 (10.8)

*Predominant types of OAB were defined as follows: urgency incontinence = urge incontinence only; mixed = mixed stress/urge incontinence with urge as a predominant factor; frequency = frequency/urgency without incontinence.

^†^% of patients who took previous OAB medication.

^‡^Patients could choose more than one reason for discontinuation of previous OAB medication or could discontinue for “other reasons” (data not shown). Thus, patients who checked both “insufficient effect” and “poor tolerability” as reasons for discontinuation of previous OAB medication could be included in both categories.

*FAS* full analysis set, *SAF* safety analysis set, *SD* standard deviation, *OAB* overactive bladder, *ER* extended release.

### Efficacy

#### Mean number of incontinence episodes/24 h by prior antimuscarinic status

**Figure 2:**
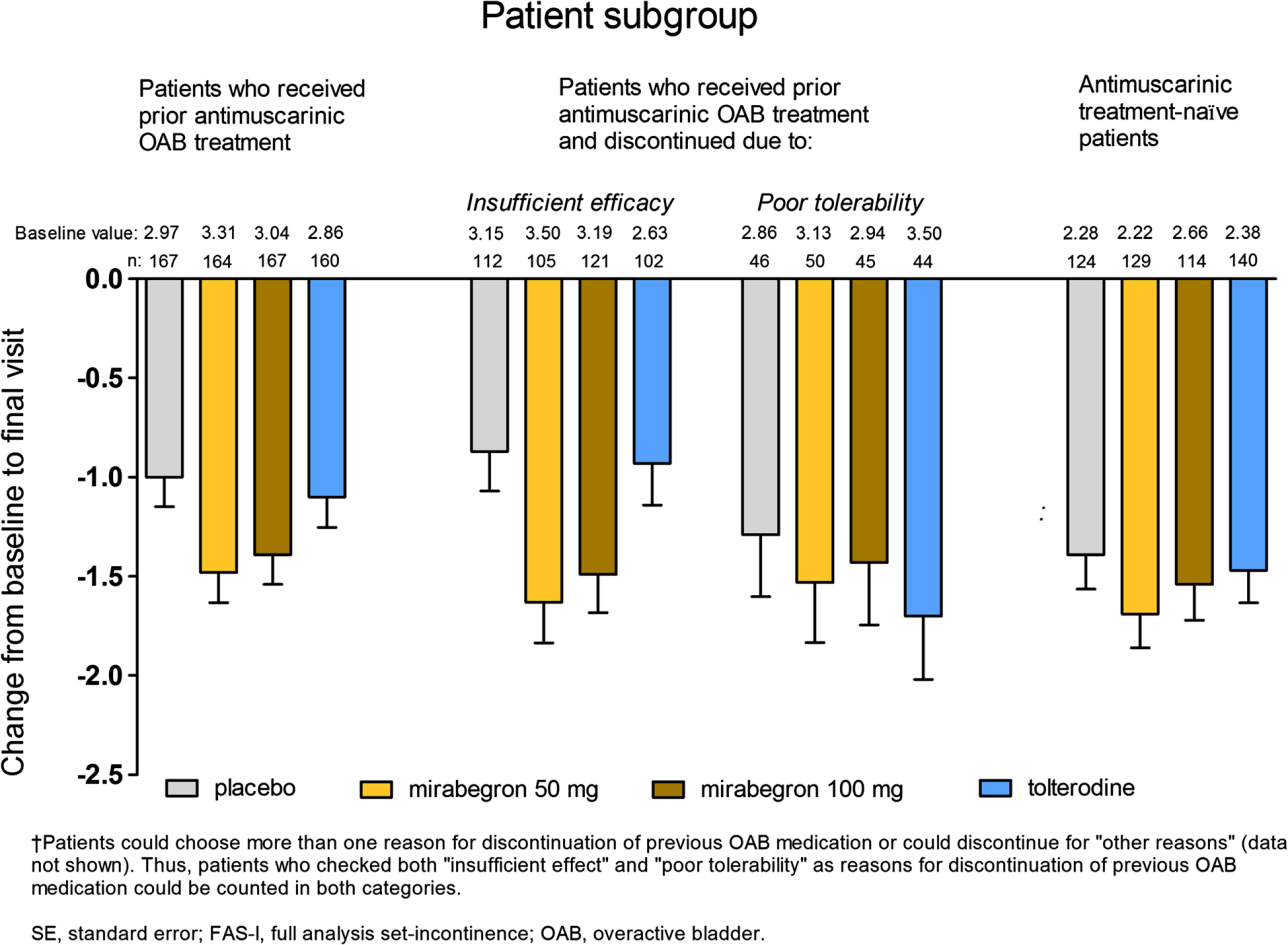
**Adjusted mean change from baseline to final visit (SE) by treatment group in mean number of incontinence episodes/24 h (FAS-I) for subgroups of patients who received prior antimuscarinic OAB medication; who discontinued it because of either insufficient efficacy or poor tolerability**^**†**^**; and for those who were antimuscarinic treatment-naïve.**

In patients who had received prior antimuscarinic OAB therapy, as well as in antimuscarinic treatment-naïve patients, both doses of mirabegron resulted in numerical improvement relative to placebo in the mean frequency of incontinence episodes (Figure 2). Adjusted mean changes from baseline to final visit (± standard error [SE]) were: –1.00 (± 0.15) for placebo; –1.48 (± 0.15) for mirabegron 50 mg; and –1.39 (± 0.15) for mirabegron 100 mg in patients who had received prior antimuscarinic therapy; and –1.39 (± 0.17) for placebo; –1.69 (± 0.17) for mirabegron 50 mg; and –1.54 (± 0.18) for mirabegron 100 mg in antimuscarinic treatment-naïve patients (Figure 2). In patients who had received prior antimuscarinic OAB therapy and in treatment-naïve patients, the magnitude of the effect of tolterodine (change from baseline to final visit [± SE] of –1.10 [± 0.15] and –1.47 [± 0.16], respectively) was lower than observed with either dose of mirabegron (Figure 2).

The placebo effect was larger in antimuscarinic treatment-naïve patients than in patients who had received prior antimuscarinic therapy (Figure 2). Thus, for both doses of mirabegron, a smaller placebo-adjusted treatment response was seen in antimuscarinic treatment-naïve patients than in those who had received prior antimuscarinic OAB therapy (Table 2).

**Table 2:** Adjusted mean difference versus placebo (95% two-sided CI) in adjusted mean change from baseline to final visit for number of incontinence episodes/24 h and number of micturitions/24 h for subgroups of patients who received prior antimuscarinic OAB medication; who discontinued it because of either insufficient efficacy or poor tolerability; and for those who were antimuscarinic treatment-naïve

	**Incontinence episodes/24 h (FAS-I)**			**Micturitions/24 h (FAS)**		
	**Mirabegron 50 mg**	**Mirabegron 100 mg**	**Tolterodine ER 4 mg**	**Mirabegron 50 mg**	**Mirabegron 100 mg**	**Tolterodine ER 4 mg**
Patient subgroup						
Received prior antimuscarinic OAB medication and discontinued	−0.48 (–0.90, –0.06)	−0.39 (–0.81, 0.02)	−0.10 (–0.52, 0.32)	−0.68 (–1.12, –0.25)	−0.51 (–0.94, –0.08)	−0.20 (–0.64, 0.23)
Discontinued* *due to:*						
Insufficient efficacy	−0.76 (–1.32, –0.19)	−0.62 (–1.16, –0.07)	−0.06 (–0.63, 0.50)	−0.59 (–1.15, –0.04)	−0.58 (–1.13, –0.02)	−0.08 (–0.64, 0.47)
Poor tolerability	−0.24 (–1.09, 0.61)	−0.14 (–1.01, 0.73)	−0.41 (–1.28, 0.46)	−0.77 (–1.64, 0.09)	−0.75 (–1.61, 0.12)	−0.18 (–1.08, 0.71)
Antimuscarinic treatment-naïve patients	−0.29 (–0.77, 0.18)	−0.15 (–0.64, 0.34)	−0.08 (–0.55, 0.39)	−0.52 (–0.95, –0.09)	−0.37 (–0.80, 0.06)	−0.29 (–0.71, 0.14)

*Based on subset of patients who had received prior OAB medication; patients could choose more than one reason for discontinuation of previous OAB medication or could discontinue for “other reasons” (data not shown). Thus, patients who checked both “insufficient effect” and “poor tolerability” as reasons for discontinuation of previous OAB medication could be included in both categories.

*FAS-I* full analysis set-incontinence, *FAS* full analysis set, *CI* confidence interval, *OAB* overactive bladder.

In patients who discontinued prior antimuscarinic medication due to insufficient efficacy, both mirabegron groups were associated with numerical improvement compared with placebo in the mean number of incontinence episodes/24 h (Figure 2). Adjusted mean changes from baseline to final visit (± SE) were: –0.87 (± 0.20) for placebo; –1.63 (± 0.21) for mirabegron 50 mg; and –1.49 (± 0.19) for mirabegron 100 mg. The corresponding change for tolterodine was –0.93 (± 0.21), resulting in a treatment effect comparable to that of placebo (adjusted difference versus placebo [95% CI] of –0.06 [–0.63, 0.50]; Table 2).

In patients who discontinued prior therapy due to poor tolerability, adjusted mean changes from baseline to final visit (± SE) were: –1.29 (± 0.31) for placebo; –1.53 (± 0.30) for mirabegron 50 mg; and –1.43 (± 0.32) for mirabegron 100 mg (Figure 2). The higher placebo response in this patient subgroup compared with the subgroup that discontinued treatment due to insufficient efficacy resulted in smaller placebo-adjusted treatment benefits with both mirabegron doses (Table 2).

#### Mean number of micturitions per 24 h by prior antimuscarinic status

**Figure 3:**
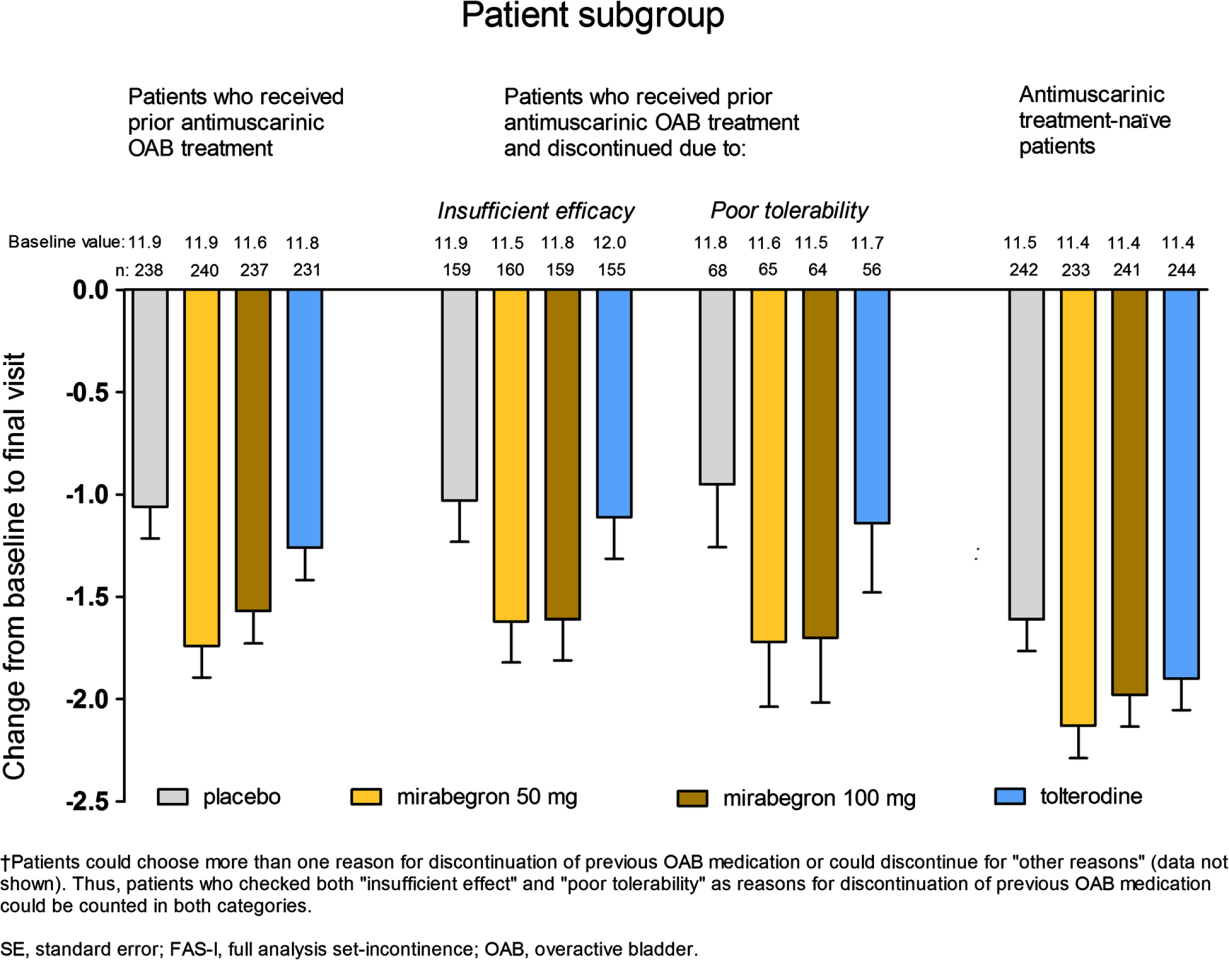
**Adjusted mean change from baseline to final visit (SE) by treatment group in mean number of micturitions/24 h (FAS) for subgroups of patients who received prior antimuscarinic OAB medication; who discontinued it because of either insufficient efficacy or poor tolerability**^**†**^**; and for those who were antimuscarinic treatment-naïve.**

In patients who received prior antimuscarinic therapy, mirabegron demonstrated numerical improvement relative to placebo in the frequency of micturitions in OAB patients. Adjusted mean changes from baseline to final visit (± SE) were: –1.06 (± 0.16) for placebo; –1.74 (± 0.16) for mirabegron 50 mg; –1.57 (± 0.16) for mirabegron 100 mg; and –1.26 (± 0.16) for tolterodine (Figure 3).

In antimuscarinic treatment-naïve patients, all active treatment groups also demonstrated numerical improvement relative to placebo in the mean number of micturitions/24 h. Adjusted mean changes from baseline to final visit (± SE) were: –1.61 (± 0.16) for placebo; –2.13 (± 0.16) for mirabegron 50 mg; –1.98 (± 0.16) for mirabegron 100 mg; and –1.90 (± 0.15) for tolterodine (Figure 3). The placebo effect was seen to be larger in treatment-naïve patients than in those who had received prior antimuscarinic therapy (Figure 3), resulting in smaller placebo-adjusted treatment benefits with both mirabegron doses in the former compared with the latter subgroup (Table 2).

In patients who discontinued prior antimuscarinic therapy due to either insufficient efficacy or poor tolerability, mirabegron showed a larger improvement from baseline than placebo, whereas the change from baseline with tolterodine was similar to that seen with placebo (Figure 3). Adjusted mean changes from baseline to final visit (± SE) were: –1.03 (± 0.20) for placebo; –1.62 (± 0.20) for mirabegron 50 mg; –1.61 (± 0.20) for mirabegron 100 mg; and –1.11 (± 0.20) for tolterodine in patients who discontinued due to insufficient efficacy and –0.95 (± 0.31) for placebo; –1.72 (± 0.32) for mirabegron 50 mg; –1.70 (± 0.32) for mirabegron 100 mg; and –1.14 (± 0.34) for tolterodine in patients who discontinued due to poor tolerability (Figure 3).

## Discussion

Antimuscarinics are considered the mainstay oral pharmacological treatment for OAB and, while effective in many OAB patients, a significant proportion of patients have a suboptimal response to treatment or experience intolerable side effects and discontinue therapy. These patients are then left with limited treatment options and may have to consider more invasive surgical procedures. For this reason, we evaluated the efficacy and tolerability of mirabegron in subpopulations of patients who were treatment-naïve or who had received prior antimuscarinic OAB therapy but discontinued because of insufficient efficacy or poor tolerability, in this post hoc analysis of a Phase 3 clinical trial conducted in Europe and Australia [17]. This analysis shows that mirabegron had a numerically positive treatment effect on incontinence and micturition frequency in patients who were treatment-naïve as well as in those who had received, but discontinued, prior antimuscarinic therapy, regardless of whether they had discontinued due to insufficient efficacy or poor tolerability.

It was notable that for each end point, the placebo effect was larger in treatment-naïve patients than in patients who had received prior antimuscarinic therapy, which is reflected in the observed treatment effect sizes. Possible factors contributing to the larger placebo effect include changes in drinking and voiding habits that result from the increased patient awareness of the disease that goes with study participation; such changes will inevitably be larger in treatment-naive patients. Moreover, the placebo effect is thought to be produced by patients’ expectation of a beneficial effect [19], which is likely to be larger in treatment-naïve patients. These observations are consistent with what has been seen in previous clinical trials of antimuscarinic agents [20].

At the time that this trial was designed, tolterodine was the most widely prescribed antimuscarinic agent for the treatment of OAB. Thus, tolterodine was considered to be the most appropriate agent to use as an active control in this trial (as in other trials in the mirabegron clinical trial program), to provide context to the efficacy and tolerability of mirabegron. Upon analysis of the study population, it emerged that of those patients in the group who had received prior antimuscarinic treatment, only 25% had previously received tolterodine. Thus, 75% of patients in the tolterodine group who had received prior antimuscarinic treatment had received antimuscarinics other than tolterodine. However, regardless of which antimuscarinic agent was discontinued due to lack of efficacy in these patients, there was no treatment benefit upon re-treatment with an antimuscarinic, in this case tolterodine, as the response to tolterodine in this subgroup of patients was similar to that seen with placebo. The fact that the effect of tolterodine on micturition frequency in antimuscarinic treatment-naïve patients was numerically similar to that seen with mirabegron supports these findings. In contrast, the antimuscarinics, solifenacin [21] and fesoterodine [22], have been shown to significantly improve OAB symptoms in OAB patients who were dissatisfied with previous tolterodine treatment. However, in contrast with our study, both studies had a flexible dose design, incorporating an optional dose increase of the antimuscarinic agent.

We acknowledge that the analysis has limitations. The study design did not allow for a head-to-head comparison of the effects of mirabegron versus tolterodine, with the latter included as an active control. The study design allowed patients to check one or more reasons for discontinuation of prior antimuscarinic medication. Thus, there was a small degree of overlap between the subgroups of patients who discontinued prior antimuscarinic treatment due to insufficient efficacy and those who discontinued due to poor tolerability. A small number of patients had discontinued due to both insufficient efficacy and poor tolerability and were therefore included in both subgroups. However, as evaluation of the study population revealed this to be a very small number of patients, post hoc analysis of this group was not conducted. No data were collected on how long patients used prior antimuscarinic therapy; as there were no restrictions on how many antimuscarinic drugs patients had used prior to the screening visit of this trial, no evaluation of treatment duration was attempted. Also, the study was not powered to detect statistically significant differences between treatment groups in each subgroup. Additionally, there are two main challenges that are inherent to post hoc subgroup analyses, namely, multiple hypothesis testing and the loss of randomization [23,24]. Multiple subgroup analyses inevitably involve multiple statistical tests, which inflate the type 1 error rate. In an effort to avoid over-interpretation of these results, we did not report p values for mean treatment differences versus placebo [24].

## Conclusions

Mirabegron provided numerical improvements in incontinence and micturition frequency in treatment-naïve patients and in patients who had received prior antimuscarinic therapy and discontinued due to insufficient efficacy or poor tolerability. In prior antimuscarinic users who discontinued due to insufficient efficacy, mirabegron showed numerical improvements in both outcomes whereas re-treatment with the antimuscarinic, tolterodine, produced an effect size similar to placebo. The efficacy and tolerability profile of mirabegron suggest that it may represent a valuable therapeutic option for patients with OAB who experience insufficient benefit from antimuscarinic therapy and in those who are intolerant of the associated AEs (e.g., dry mouth, constipation). In patients who received mirabegron in the overall trial, dry mouth, the most common [6,7] and bothersome side effect of antimuscarinic agents [8], occurred with a similar incidence as with placebo (2.6–2.8%) and a three-fold lower incidence than in patients receiving tolterodine ER 4 mg (10.1%). This post hoc analysis provides valuable insights but confirmation of its results will be required in randomized prospective trials of OAB patients with and without prior antimuscarinic therapy.

### Consent

All patients provided written informed consent.

## Abbreviations

OAB: Overactive bladder; AEs: Adverse events; CI: Confidence intervals; eCRF: Electronic case report form; ER: Extended release; SAF: Safety analysis set; FAS: Full analysis set; FAS-I: Full analysis set – incontinence; SD: Standard deviation; SE: Standard error; ER: Slow release.

## Competing interests

Vik Khullar has received support for travel from Astellas; payment for lectures, including service on speakers’ bureaus, from Astellas and Pfizer; payment for development of education presentations from Pfizer; and fees for board membership and consultancy from Astellas, Pfizer, and Allergan. In addition, his institution has received grants or has grants pending from Astellas and Pfizer. Javier Cambonero has acted as a principal investigator in the clinical trials that are the subject of the submitted work; he has no conflicts of interest to declare. Javier Angulo has received payment for lectures including service at speakers’ bureaus from Astellas, Pfizer, and GSK. His institution has received grants or has grants pending from Astellas and Pfizer. Mary Beth Blauwet, Caroline Dorrepaal, and Marianne Wooning are employees of the study sponsor (Astellas) and Nancy Martin is a former employee of the study sponsor (Astellas); none have any other relationships that represent a conflict of interest.

## Authors’ contributions

JA CD, MW, VK, NM and MBB were involved in the conception and design of the study; JA, VK and JC were responsible for the acquisition of data; and JA, CD, MW, VK, NM, JC and MBB analysed and interpreted the data. NM and MBB were responsible for the statistical analysis of the data. All the authors were involved in drafting the manuscript and in revising it critically for important intellectual content. All authors read and approved the final manuscript.

## Pre-publication history

The pre-publication history for this paper can be accessed here:

http://www.biomedcentral.com/1471-2490/13/45/prepub

## References

[B1] Irwin DE Kopp ZS Agatep B Milsom I Abrams P Worldwide prevalence estimates of lower urinary tract symptoms, overactive bladder, urinary incontinence and bladder outlet obstruction BJU Int 2011 108 1132 1138 10.1111/j.1464-410X.2010.09993.x 21231991

[B2] Chapple C Cruz F Open to debate. The Motion: antimuscarinics are the mainstay of therapy for overactive bladder Eur Urol 2008 54 226 230 18439750 10.1016/j.eururo.2008.04.020

[B3] Chapple CR Khullar V Gabriel Z Muston D Bitoun CE Weinstein D The effects of antimuscarinic treatments in overactive bladder: an update of a systematic review and meta-analysis Eur Urol 2008 54 543 562 10.1016/j.eururo.2008.06.047 18599186

[B4] Yamaguchi O Nishizawa O Takeda M Yokoyama O Homma Y Kakizaki H Obara K Gotoh M Igawa Y Seki N Yoshida M Clinical guidelines for overactive bladder Int J Urol 2009 16 126 142 10.1111/j.1442-2042.2008.02177.x 19228224

[B5] Abrams P Andersson KE Muscarinic receptor antagonists for overactive bladder BJU Int 2007 100 987 1006 10.1111/j.1464-410X.2007.07205.x 17922784

[B6] Kessler TM Bachmann LM Minder C Löhrer D Umbehr M Schünemann HJ Kessels AG Adverse event assessment of antimuscarinics for treating overactive bladder: a network meta-analytic approach PLoS One 2011 6 e16718 10.1371/journal.pone.0016718 21373193 PMC3044140

[B7] Chapple C Khullar V Gabriel Z Dooley JA The effects of antimuscarinic treatments in overactive bladder: a systematic review and meta-analysis Eur Urol 2005 48 5 26 10.1016/j.eururo.2005.02.024 15885877

[B8] Benner JS Nichol MB Rovner ES Jumadilova Z Alvir J Hussein M Fanning K Trocio JN Brubaker L Patient-reported reasons for discontinuing overactive bladder medication BJU Int 2010 105 1276 1282 10.1111/j.1464-410X.2009.09036.x 19912188

[B9] D’Souza AO Smith MJ Miller LA Doyle J Ariely R Persistence, adherence, and switch rates among extended-release and immediate-release overactive bladder medications in a regional managed care plan J Manag Care Pharm 2008 14 291 301 18439051 10.18553/jmcp.2008.14.3.291 PMC10438114

[B10] Sexton CC Notte SM Maroulis C Dmochowski RR Cardozo L Subramanian D Coyne KS Persistence and adherence in the treatment of overactive bladder syndrome with anticholinergic therapy: a systematic review of the literature Int J Clin Pract 2011 65 567 585 10.1111/j.1742-1241.2010.02626.x 21489081

[B11] Shaya FT Blume S Gu A Zyczynski T Jumadilova Z Persistence with overactive bladder pharmacotherapy in a Medicaid population Am J Manag Care 2005 11 S121 S129 16161385

[B12] Wagg A Compion G Fahey A Siddiqui E Persistence with prescribed antimuscarinic therapy for overactive bladder: a UK experience BJU Int 2012 110 1767 1774 10.1111/j.1464-410X.2012.11023.x 22409769

[B13] Takasu T Ukai M Sato S Matsui T Nagase I Maruyama T Sasamata M Miyata K Uchida H Yamaguchi O Effect of (*R*)-2-(2-aminothiazol-4-yl)-4′-{2-[(2-hydroxy-2-phenylethyl)amino]ethyl} acetanilide (YM178), a novel selective b_3_-adrenoceptor agonist, on bladder function J Pharmacol Exp Ther 2007 321 642 647 10.1124/jpet.106.115840 17293563

[B14] Kumar V Templeman L Chapple CR Chess-Williams R Recent developments in the management of detrusor overactivity Curr Opin Urol 2003 13 285 291 10.1097/00042307-200307000-00004 12811292

[B15] Yamaguchi O β3-adrenoceptors in human detrusor muscle Urology 2002 59 25 29 12007519 10.1016/s0090-4295(01)01635-1

[B16] Nitti VW Auerbach S Martin N Calhoun A Lee M Herschorn S Results of a randomized phase III trial of mirabegron in patients with overactive bladder J Urol 2012 189 1388 1395 23079373 10.1016/j.juro.2012.10.017

[B17] Khullar V Amarenco G Angulo J Cambronero J Høye K Milsom I Radziszewski P Rechberger T Boerrigter P Drogendijk T Efficacy and tolerability of mirabegron, a β(3)-adrenoceptor agonist, in patients with overactive bladder: results from a randomised European-Australian phase 3 trial Eur Urol 2013 63 283 295 10.1016/j.eururo.2012.10.016 23182126

[B18] Van Kerrebroeck P Barkin J Castro-Diaz D Espuña-Pons M Frankel J Gousse A Martin N Stolzel M Gunther A Herschorn S Randomised, double-blind, placebo-controlled Phase III study to assess the efficacy and safety of mirabegron 25 mg and 50 mg once-daily in overactive bladder (OAB) 2012 Beijing, China: International Continence Society

[B19] Brody H Miller FG Lessons from recent research about the placebo effect–from art to science JAMA 2011 306 2612 2613 10.1001/jama.2011.1850 22187283

[B20] van Leeuwen JH Castro R Busse M Bemelmans BL The placebo effect in the pharmacologic treatment of patients with lower urinary tract symptoms Eur Urol 2006 50 440 452 discussion 453 10.1016/j.eururo.2006.05.014 16753253

[B21] Chancellor MB Zinner N Whitmore K Kobashi K Snyder JA Siami P Karram M Laramée C Capo JP Jr Seifeldin R Efficacy of solifenacin in patients previously treated with tolterodine extended release 4 mg: results of a 12-week, multicenter, open-label, flexible-dose study Clin Ther 2008 30 1766 1781 10.1016/j.clinthera.2008.10.011 19014833

[B22] Wyndaele JJ Goldfischer ER Morrow JD Gong J Tseng LJ Guan Z Choo MS Effects of flexible-dose fesoterodine on overactive bladder symptoms and treatment satisfaction: an open-label study Int J Clin Pract 2009 63 560 567 10.1111/j.1742-1241.2009.02035.x 19348029 PMC2705818

[B23] Magee CD Byars LA DeZee KJ Limitations of subgroup analyses in meta-analysis of cardiac resynchronization therapy by QRS duration Arch Intern Med 2012 172 375 376 22371934 10.1001/archinternmed.2011.1499

[B24] Lagakos SW The challenge of subgroup analyses–reporting without distorting N Engl J Med 2006 354 1667 1669 10.1056/NEJMp068070 16625007

